# Notoginsenoside R1 modulates the ATF4-CHOP-Bcl2 signaling pathway to alleviate mitochondrial stress damage and apoptosis in asthmatic airway epithelial cells

**DOI:** 10.3389/fimmu.2026.1861285

**Published:** 2026-09-28

**Authors:** Yaozheng Ling, Ling Guan, Yu Zhao, Zhou Fu, Wenjing Zou

**Affiliations:** 1 Department of Respiratory Medicine Children’s Hospital of Chongqing Medical University, National Clinical Research Center for Children and Adolescents’ Health and Diseases, Ministry of Education Key Laboratory of Child Development and Disorders, Chongqing, China; 2 Chongqing Key Laboratory of Pediatric Metabolism and Inflammatory Diseases, Chongqing, China

**Keywords:** airway epithelial damage, asthma, ATF4-CHOP-Bc2 signaling pathway, mitochondrial stress damage, notoginsenoside R1

## Abstract

**Objective:**

To investigate whether Notoginsenoside R1 (NG-R1) improves mitochondrial homeostasis by targeting the integrin signaling response (ISR) core transcription factor ATF4, thereby alleviating damage and apoptosis in asthma airway epithelial cells.

**Methods:**

House dust mites (HDM) were used to establish an *in vivo* asthma mouse model and an *in vitro* BEAS-2B cell model. The anti-inflammatory and immunomodulatory effects of NG-R1 were evaluated through pulmonary function testing, bronchoalveolar lavage fluid (BALF) cell counting, and hematoxylin and eosin (H&E) staining. We evaluated the effects of NG-R1 on apoptosis and mitochondrial function using TUNEL, Annexin V-FITC/PI staining, transmission electron microscopy, MitoSox Red, TMRE, JC-1, and Western blot analysis of mitochondrial dynamics-related proteins (p-Drp1/Drp1, Mfn2, ATP5F1A). We used lentiviral knockdown of ATF4 combined with RNA-seq, GO enrichment analysis, and the STRING database to predict its downstream mechanisms. Molecular docking was used to predict the binding affinity between NG-R1 and ATF4, and the expression levels of ATF4, CHOP, and Bcl-2 were validated using Western blot analysis in both *in vivo* and *in vitro* models.

**Results:**

ATF4 expression was significantly elevated in lung tissue from asthmatic mice and in HDM-stimulated BEAS-2B cells, and was positively correlated with increased epithelial cell apoptosis and reduced migration and repair capacity. ATF4 knockdown alleviates HDM-induced increases in mitochondrial reactive oxygen species (ROS), decreases in membrane potential, mitochondrial ultrastructural damage, and disbalance in fission/fusion (downregulation of p-Drp1/Drp1 and upregulation of Mfn2 and ATP5F1A) via the ATF4-CHOP-Bcl2 signaling pathway, thereby inhibiting apoptosis. NG-R1 intervention significantly reduced airway hyperresponsiveness, total inflammatory cell counts in BALF, and levels of Th2 cytokines (IL-4, IL-13, IL-33) in asthmatic mice, and alleviated pulmonary inflammatory infiltration. Molecular docking and *in vitro* and *in vivo* experiments indicated that NG-R1 may specifically downregulate ATF4 (binding energies of -5.761 and -6.553 kcal/mol, respectively), modulate CHOP-Bcl2 expression levels, improve mitochondrial function and dynamic equilibrium, inhibit epithelial cell apoptosis, and promote migration and repair.

**Conclusion:**

NG-R1 may regulate the ATF4-CHOP-Bcl2 signaling pathway by specifically binding to and downregulating ATF4, thereby alleviating mitochondrial stress-induced damage, inhibiting airway epithelial cell apoptosis, and promoting epithelial repair, suggesting that NG-R1 is a potential candidate drug for targeted asthma therapy.

## Introduction

Asthma is a common chronic inflammatory airway disease that affects approximately 330 million people worldwide. Its prevalence varies considerably across regions, ranging from 0.9% to 29.0%, and has been increasing by approximately 50% per decade (1, 2). Asthma can be classified into distinct subtypes according to the types of inflammatory cells infiltrating the airways. Although these subtypes differ in their inflammatory phenotypes, airway epithelial damage represents a common pathological feature (3). As the first line of defense against allergens, inhaled irritants, and pathogens, the airway epithelium functions as both a physical and an immune barrier. When airway epithelial cells are exposed to external stimuli such as allergens and gaseous pollutants, the intracellular production of oxidative stress mediators increases, including reactive oxygen species and reactive nitrogen species, leading to cellular stress, damage, and even apoptosis (4). Damage to the epithelial barrier enables external irritants to penetrate the damaged epithelium and reach the submucosal layer, where they excessively activate epithelial cell signaling cascades and promote the recruitment and activation of immune cells, such as macrophages and leukocytes, thereby inducing local inflammation and immune responses. Prolonged and persistent inflammatory stimulation further contributes to airway epithelial shedding, goblet cell hyperplasia, subepithelial fibrosis, and hypertrophy and hyperplasia of airway smooth muscle cells, ultimately driving the formation and progression of airway remodeling. This pathological process narrows the airway lumen in patients with asthma and exacerbates clinical symptoms, including chest tightness and dyspnea (5). Collectively, these findings indicate that damage to airway epithelial cells plays a critical role in the pathogenesis of allergic asthma.

When the lungs are exposed to external stressors, their ability to respond largely depends on the coordinated activation of stress response proteins. In this context, the integrated stress response (ISR) pathway, an evolutionarily highly conserved cellular signaling network, constitutes a key adaptive mechanism through which lung tissue responds to environmental challenges (6). The ISR is mediated by one or more of four stress-responsive kinases: GCN2 (EIF2AK4), PKR (EIF2AK2), PERK (EIF2AK3), and HRI (EIF2AK1). Upon sensing diverse stress signals, these kinases phosphorylate eukaryotic initiation factor 2α (eIF2α), which in turn upregulates the expression of activating transcription factor 4 (ATF4). As a central effector of the ISR pathway, ATF4 plays a critical role in regulating the expression of downstream genes under various stress conditions, including endoplasmic reticulum stress, amino acid deprivation, mitochondrial stress, and oxidative stress, among others.

In recent years, there has been growing recognition of the central role played by dysregulation of intracellular organelles, particularly mitochondrial dysfunction, in the pathophysiology of various diseases (7). As the key hubs of cellular energy metabolism, mitochondria supply ATP through oxidative phosphorylation. Maintaining the dynamic balance between their morphology and function, which is precisely regulated by processes such as fusion, fission, biogenesis, and mitophagy, is essential for cellular homeostasis (8). Under conditions of increased cellular metabolic demand or pathological states, mitochondria undergo adaptive morphological changes, with mitochondrial fission often playing a dominant role in response to energy stress or damage (9). When mitochondrial dysfunction occurs as result of external factors, it can trigger a series of pathological alterations, including an imbalance in the mitochondrial oxidative and antioxidant systems, mitochondrial DNA (mtDNA) damage, disrupted mitochondrial dynamics, and abnormal mitophagy. These changes lead to excessive production of reactive oxygen species (ROS) within mitochondria and the cell, thereby inducing oxidative stress and ultimately resulting in cellular damage or death (10). Studies have shown that significant mitochondrial oxidative stress is present in the airway epithelium of patients with asthma, and this stress directly influences epithelial cell damage and apoptosis (11). Throughout the course of asthma, airway epithelial cells are chronically exposed to external stimuli such as allergens, particulate matter, and pathogenic microorganisms, leading to sustained accumulation of ROS within both the cells and mitochondria, which directly or indirectly damages the respiratory epithelium. Given the close interplay between inflammation and oxidative stress, tissue damage induced by oxidative stress can further activate inflammatory and immune responses, which in turn exacerbate oxidative stress levels, creating a vicious cycle that drives asthma progression (12).

Notoginsenoside R1 (NG-R1) is the primary active saponin component extracted from the dried roots and rhizomes of Sanqi. Studies have demonstrated that NG-R1 exerts a wide range of pharmacological effects in cardiovascular protection, neuroprotection, and immune regulation by inhibiting oxidative damage, fibrosis, inflammatory responses, apoptosis, and endoplasmic reticulum stress (13). The single antioxidant compound has been shown to be ineffective in the treatment of asthma (11). However, NG-R1, a multi-targeted natural product with anti-inflammatory, antioxidant, and immunomodulatory properties, is attracting increasing attention as a potential therapeutic agent for asthma. Given the pivotal role of mitochondrial stress and airway epithelial damage in the pathogenesis of asthma, investigating whether NG-R1 can improve mitochondrial homeostasis by regulating ATF4, a core transcription factor in the ISR, and thereby alleviate airway epithelial cell damage and apoptosis will be an important direction for future research into targeted asthma therapies.

In this study, we utilized House Dust Mite (HDM) to establish an *in vivo* asthma model in mice and an *in vitro* asthma model using BEAS-2B cells to investigate the relationship between ATF4 expression and asthmatic airway epithelial injury and apoptosis. Through RNA-seq, Gene Ontology (GO) enrichment analysis, and protein–protein interaction (PPI) network analysis, we identified ATF4-regulated signaling pathways associated with mitochondrial stress, epithelial cell damage, and apoptosis. Furthermore, using both *in vitro* and *in vivo* experiments, we validated the anti-inflammatory and immunomodulatory effects of NG-R1 in asthma, as well as its targeted regulation of the ATF4-CHOP-Bcl2 signaling pathway. Our aim is to elucidate the mechanism of action of NG-R1 in ameliorating airway epithelial damage in asthma, thereby providing experimental evidence to support its clinical application and establishing a foundation for targeted asthma therapy.

## Materials and methods

### Animals

Female C57BL/6 mice, aged 6–8 weeks and weighing approximately 20 g, were purchased from Chengdu Jicui Yaokang Biotechnology Co., Ltd. (Chengdu, China). The mice were housed in the specific pathogen-free (SPF) facility at the Laboratory Animal Center of the Children’s Hospital Affiliated to Chongqing Medical University (Chongqing, China) under controlled conditions: ambient temperature of 22–26 °C, humidity of 55%–60%, and a 12-hour light/dark cycle. All animal experiments were conducted in accordance with the guidelines of the Institutional Animal Care and Use Committee (IACUC) and were approved by the Ethics Committee of Chongqing Medical University (Approval No. CHCMU-IACUC20251010002).

### Cell culture

BEAS-2B cells were purchased from Haixing Biotechnology (Suzhou, China) and cultured in high-glucose DMEM (Gibco, USA, 6126013) supplemented with 10% fetal bovine serum (Sigma-Aldrich, Australia, F8687) at 37 °C in a 5% CO_2_ incubator. Based on the results of preliminary concentration gradient experiments, BEAS-2B cells were treated with HDM (Greer Labs, USA, XPB82D3A2.5) at a concentration of 50 μg/ml for 24 hours to establish an *in vitro* asthma model. In addition to HDM intervention, BEAS-2B cells were also treated with dexamethasone (Dex) (MCE, USA, HY-B1829A), NG-R1 (Macklin, Shanghai, China, N814983), or a combination of both, each at a concentration of 5 mM. The administration concentrations of Dex and NG-R1 were determined based on concentration gradient pre-experiments.

### Lentiviral transfection

Cell transfection was performed using lentiviruses LV-GFP and LV-shATF4, which were constructed by Shanghai Genechem Co., Ltd. and carried the puromycin resistance gene. BEAS-2B cells were seeded in a 6-well plate and allowed to grow and adhere overnight in an incubator. Once the confluence reached 50%–60%, the required volume of lentivirus was calculated based on a multiplicity of infection (MOI) of 100. At the same time, transfection reagent was added at a ratio of 4 μL per 100 μL of culture medium, and the mixture was gently mixed until uniform. The mixture was then added to the 6-well plate, and the cells were cultured for 12–16 hours. Subsequently, the medium was replaced with fresh complete medium, and culture was continued. Starting on the fourth day post-transfection, green fluorescent protein (GFP) expression in the transfected cells could be observed under a fluorescence microscope, and transfection efficiency was calculated by analyzing the percentage of GFP-positive cells using flow cytometry.

### Establishment of asthma mouse model and intervention

The mouse asthma model was established according to the protocol described in previous studies (14), the procedure is shown in **Figure 1A**. Mice were intranasally sensitized with HDM (20 μg) on days 0, 7, and 14, followed by intranasal challenge with HDM (20 μg) on days 21, 23, 25, 27, and 29. Mice in the control group were sensitized and challenged with saline instead of HDM. All mice were anesthetized via intraperitoneal injection of 1.25% tribromoethanol (25 μL/g) prior to intranasal sensitization and challenge. In the intervention groups, mice received intranasal challenges on days 21, 23, 25, 27, and 29, accompanied by intraperitoneal injections of dexamethasone (Dex; 5 mg/kg), NG-R1 (5 mg/kg), or a combination of Dex and NG-R1 (5 mg/kg). Lung function was assessed on day 30, and all mice were euthanized with an overdose of anesthetic (1.25% tribromoethanol, 40 μL/g, administered intraperitoneally) for tissue collection on day 31, to be used in subsequent experiments and analyses.

### Lung function assessment

Mouse pulmonary function testing was performed using a mouse pulmonary function analyzer (Emka Technologies, France). After installing the nebulizer and volume recording device, the low and high curves were calibrated using the calibration program. Once calibration was completed, baseline measurements were obtained, followed by measurements after nebulization of normal saline and sequentially increasing concentrations of methacholine (Sigma-Aldrich, Australia, A2251) at 3.125, 6.25, 12.5, 25, and 50 mg/mL. Each nebulization lasted 3–5 minutes, and Penh was recorded for at least 3 minutes. After data recording, the data were exported, and the mice were returned to their cages.

### Cell count in bronchoalveolar lavage fluid

Bronchoalveolar lavage was performed on the right lung of mice three times using pre-chilled PBS. The bronchoalveolar lavage fluid (BALF) was centrifuged, and the cell pellet was collected. Erythrocytes were lysed using erythrocyte lysis buffer, and total cells were counted using a cell counter. The remaining cells were stained with Wright-Giemsa solution (Nanjing Jiancheng Bioengineering Research, Nanjing, China, D010-1-1) and counted. At least 200 cells were counted per smear, and the numbers and percentages of eosinophils, neutrophils, and lymphocytes were recorded separately.

### H&E staining of lung tissue and inflammation scoring

The left lung lobe of each mouse was fixed in 4% paraformaldehyde for at least 48 hours, then dehydrated and embedded in paraffin to obtain 4-μm-thick sections. Sections were deparaffinized with xylene, rehydrated through a graded ethanol series, stained with hematoxylin and eosin, dehydrated again through a graded ethanol series, cleared with xylene, and finally mounted with neutral resin. Lung tissue sections were examined under an optical microscope by two independent observers, who recorded pathological changes. Five fields of view per mouse lung section were evaluated, including areas containing blood vessels, airways, and pulmonary interstitium. Three scores were recorded for each field, and the sum of these three scores constituted the score for that field. The mean score was calculated by dividing the total score from the five fields by 5, yielding the pathological score for lung inflammation for that mouse. The scoring criteria for inflammation in lung tissue are as shown in **Table 1**. 

**Table 1:** Pathological scoring criteria for lung tissue.

Inflammation around the trachea and bronchioles	Perivascular inflammation	Pulmonary interstitial inflammation	Score
non-invasive	non-invasive	non-invasive	0
<2 cell thicknesses	<4 cell thicknesses	Infiltration without thickening	1
3–5 cell thicknesses	5–7 cell thicknesses	Significant infiltration and mild thickening	2
>5 cell thicknesses	>7 cell thicknesses	Significant infiltration and marked thickening	3

### Immunofluorescence

After dewaxing and rehydrating, the lung tissue sections were permeabilized with Proteinase K (20 µg/mL). Subsequently, the sections were covered with the TUNEL detection solution (MeilunBio, Dalian, China, MA0224) and incubated in the dark. Following this, DAPI staining (Beyotime, Shanghai, China, C1006) was performed. Immediately after staining, the sections were mounted with antifade mounting medium (Beyotime, Shanghai, China, P0131) and imaged using a laser confocal microscope (Nikon, Tokyo, Japan).

MitoSox Red (MCE, USA, HY-D1055) was used to label mitochondrial superoxide in BEAS-2B cells. Images were acquired using a laser confocal microscope (Nikon, Tokyo, Japan). Mitochondrial membrane potential was assessed using TMRE (Beyotime, Shanghai, China, C2001S) and JC-1 (Solarbio, Beijing, China, M8650); images were acquired with the same laser confocal microscope and analyzed with corresponding software. Apoptosis was detected using Annexin V-FITC/PI (Solarbio, Beijing, China, CA1020) and Annexin V-Cy5 (APExBIO, USA, K2005), and images were captured using the same confocal microscope.

### Enzyme-linked immunosorbent assay

To measure the levels of IL-4, IL-13, and IL-33 in mouse BALF and serum IgE, ELISA kits (IL-4: Multi Sciences, Hangzhou, China, EK204; IL-13: Multi Sciences, Hangzhou, China, EK213; IL-33: Multi Sciences, Hangzhou, China, EK233; IgE: Ruixin, Quanzhou, China, RXM202737M) were used according to the manufacturer’s instructions.

### Electron microscopy analysis

Fresh mouse lung tissue was excised, rinsed with PBS, and cut into small tissue blocks, which were immediately placed in 4% glutaraldehyde and fixed at room temperature for 2 hours. The specimens were then postfixed, dehydrated, dried, and sputter-coated with gold, and images were captured using a scanning electron microscope (Hitachi, Tokyo, Japan). For transmission electron microscopy, mouse lung tissue fixed in 4% glutaraldehyde was dehydrated, embedded, polymerized, sectioned, and stained; images were then captured using a transmission electron microscope (Hitachi, Tokyo, Japan).

BEAS-2B cells were collected, fixed with 4% glutaraldehyde, and processed following the same dehydration and embedding protocol as used for lung tissue. Finally, images were acquired using a transmission electron microscope (Hitachi, Tokyo, Japan) with samples placed on copper grids.

### Flow cytometry analysis

The efficiency of lentiviral transfection was assessed as the percentage of GFP-positive BEAS-2B cells via flow cytometry. Untreated and transfected BEAS-2B cells were collected, centrifuged, and washed separately. After resuspension in 250 μL of PBS, the percentage of GFP-positive cells was determined by flow cytometry.

### Quantitative real-time PCR

Total RNA was extracted from BEAS-2B cells using an RNA extraction kit (Bioer Technology, Hangzhou, China, BSC52M1). The mRNA was then reverse-transcribed into cDNA using a reverse transcription kit (Accurate Biology, Changsha, China, AG11728), followed by quantitative real-time PCR (qPCR) using a kit from the same manufacturer (Accurate Biology, Changsha, China, AG11701). GAPDH served as the internal reference, and relative gene expression levels were calculated using the 2^-ΔΔCt^ method. The primer sequences used for amplification are listed in **Table 2**.

**Table 2:** Primer sequences.

Primer name	Sequences (5’- 3’)
H-ATF4-F	GGCCAAGCACTTCAAACCTC
H-ATF4-R	GACTGACCAACCCATCCACA
H-GAPDH-F	GCACCGTCAAGGCTGAGAAC
H-GAPDH-R	TGGTGAAGACGCCAGTGGA

### Western blot

Protein concentrations were determined using the BCA method. Equal volumes of the samples were subjected to electrophoresis and then transferred to a PVDF membrane. After blocking with 5% skim milk for 1 hour, the membrane was incubated overnight at 4 °C with primary antibodies against the following targets: ATF4 (diluted 1:1000, ZEN-BIO, China, R381402), p-Drp1 (diluted 1:1000, HUABIO, China, HA722760), ATP5F1A (diluted 1:1000, ZEN-BIO, China, R381760), Drp1 (diluted 1:1000, ZEN-BIO, China, R30028), Bcl2 (diluted 1:1000, Abways, China, CY5032), CHOP (diluted 1:1000, ABCEPTA, China, AP11955B), and GAPDH (diluted 1:5000, Proteintech, China, 10494-1-AP). Following primary antibody incubation, the membrane was incubated with HRP-conjugated goat anti-rabbit IgG (diluted 1:1000, Beyotime, Shanghai, China, A0208) or goat anti-mouse IgG (diluted 1:1000, Beyotime, Shanghai, China, A0216) at room temperature for 1 hour. Chemiluminescent signals were detected using ECL reagent (MeilunBio, Dalian, China, MA0187), and quantitative analysis was performed using ImageJ software.

### RNA-seq and bioinformatics analysis

BEAS-2B cells that were growing well after transfection were divided into the LV-GFP + HDM group (n=3) and the LV-shATF4 group (n=3). To each group, 1mL of TRIzol lysis buffer was added for lysis. After thorough lysis, the lysate was transferred to nuclease-free Eppendorf tubes and stored at −80°C. The frozen samples were shipped via dry ice to Shanghai Meiji Biotechnology Co., Ltd. (Shanghai, China) for library preparation and sequencing. RNA was extracted using QIAzol Lysis Reagent, purified using the RNA Purification Kit, and its integrity was assessed via agarose gel electrophoresis. The RQN value was determined using the Agilent 5300. The total amount of RNA required for a single library construction was 1μg, with a concentration ≥30ng/μL and an RQN >6.5. mRNA was enriched using oligo dT, followed by the addition of a fragmentation buffer to randomly cleave the mRNA into fragments of approximately 300bp. Reverse transcriptase was then added to reverse transcribe the fragments into double-stranded structures. After adding the End Repair Mix to blunt the ends and attach adapter sequences, purification and fragment selection were performed, followed by PCR amplification to obtain the final library. Sequencing was performed on the NovaSeq X Plus platform (using Qubit 4.0 for quantification; bridge PCR amplification was carried out on the cBot to generate clusters, which were then loaded onto the sequencer). After sequencing was completed, raw sequence data were analyzed and quality-controlled (using fastp), and the data were aligned to the reference genome using HiSat2 software. Subsequent expression analysis and differential expression analysis were performed using RSEM, DEGseq, and Deseq2 software. GO analysis of differentially expressed genes was conducted online via the Bioinformatics website, and protein-protein interaction analysis was finally performed on the STRING website.

### Molecular docking

The protein structures of mouse ATF4 (Q06507) and human ATF4 (P18848) used for docking were downloaded from the AlphaFold database, while the 3D/2D structure of the NG-R1 small molecule was obtained from the PubChem database. Energy minimization was performed using the MMFF94 force field. Molecular docking was performed using AutoDock Vina (version 1.2.3). Prior to docking, the receptor protein was prepared by adding hydrogens using PyMOL (version 2.5.5). The docking box was then defined to encompass the entire protein structure. In addition, ADFRsuite (version 1.03) was used to convert the preprocessed small molecules and receptor proteins into the PDBQT format required for AutoDock Vina. During docking, the exhaustiveness of the global search was set to 32, while all other parameters were left at their default settings. The docking conformation with the highest score was considered the binding conformation. The results were visualized and analyzed using PyMOL (version 2.5.5) and Discovery Studio (version 2025).

### Statistical analysis

Each experiment was repeated at least three times. Statistical analyses and graphical representations were performed using GraphPad Prism (version 8.0). Group comparisons were assessed using Student’s t-test, one-way ANOVA, or two-way ANOVA with the Bonferroni *post hoc* test, as appropriate. Results are presented as mean ± standard error of the mean (SEM). A *P*-value < 0.05 was considered statistically significant.

## Results

### ATF4 protein expression levels are positively correlated with apoptosis in damaged airway epithelial cells in asthma

Total proteins were extracted from the lung tissues of asthmatic mice and analyzed by Western blot (**Figures 1B, C**). Compared with the control group, ATF4 protein expression was significantly upregulated in the lung tissue of the asthma group (*P* < 0.001). Similarly, in the *in vitro* asthma cell model established using BEAS-2B cells treated with HDM (**Figures 1G, H**), ATF4 expression was also increased following HDM treatment compared with control group (*P* < 0.01). The integrity of the bronchial epithelium was assessed by scanning electron microscopy (**Figure 1D**). In the control group, the bronchial cilia were neatly arranged and morphologically intact, no obvious epithelial damage or mucus-like secretions were observed in the lumen. In contrast, in the asthmatic group, bronchial mucus secretion was significantly increased, with cilia exhibiting marked flattening, sparseness, shedding, and defects, and local epithelial surfaces showing evident damage. TUNEL apoptosis assays (**Figures 1E, F**) revealed that asthmatic mice had significantly increased apoptotic signals in the airway epithelium compared with control mice (*P* < 0.001). In the *in vitro* asthma cell model, apoptosis was assessed using Annexin V-FITC/PI double staining (**Figures 1I, M, N**). Compared with the control group, apoptosis was significantly increased in BEAS-2B cells following HDM treatment (*P* < 0.01). Subsequently, a cell migration assay was performed to assess cell migration capacity (**Figures 1G, K, L**). The migration rates of cells in the HDM group were significantly lower than those in the control group at both 24h and 48h (*P* < 0.001 and *P* < 0.01, respectively), indicating that HDM treatment inhibits the migration capacity of BEAS-2B cells and thus affects epithelial repair. The results above indicate that ATF4 is highly expressed in asthma airway epithelial cells and is closely associated with epithelial cell apoptosis.

**Figure 1:**
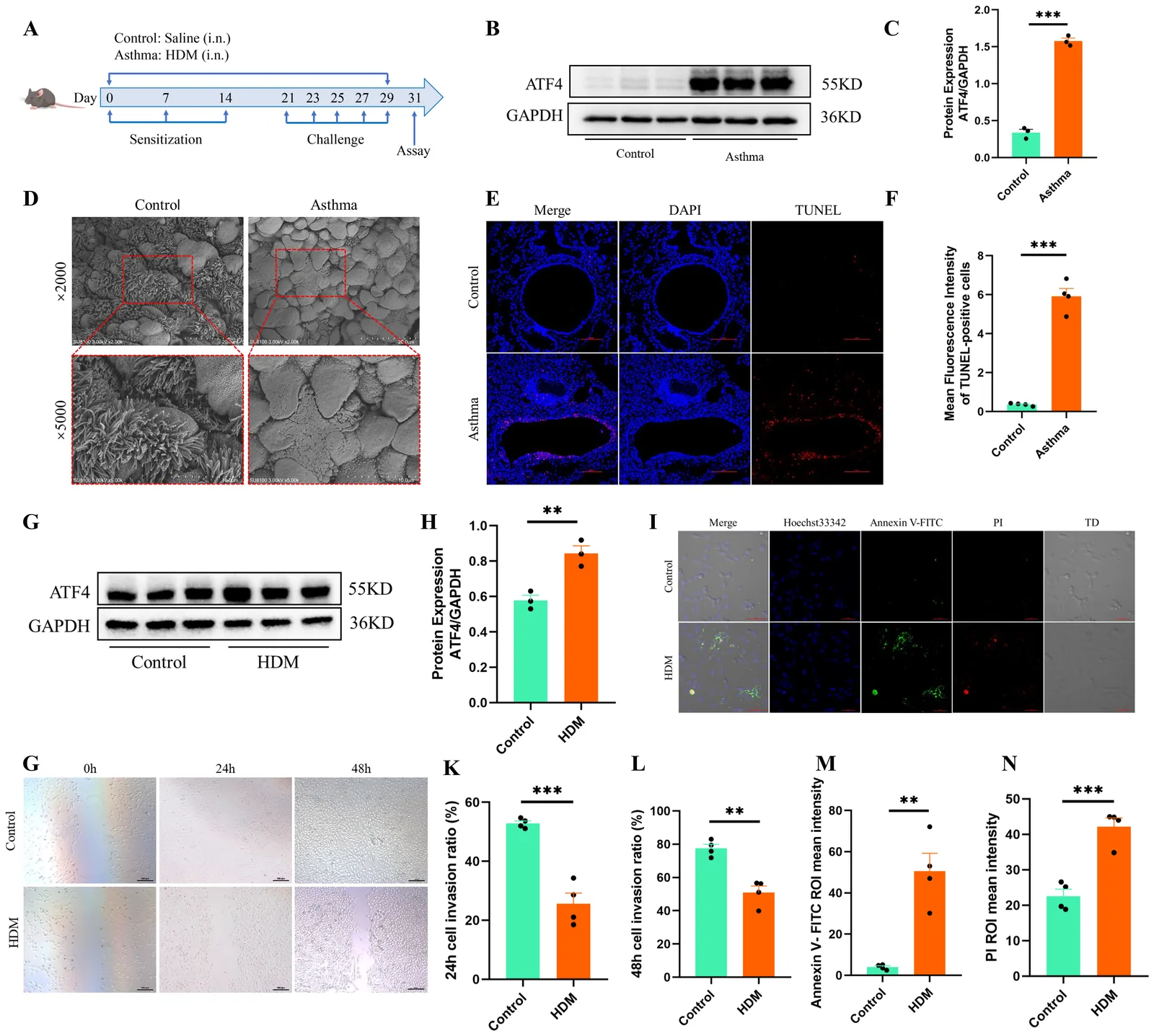
*In vitro* and *in vivo* experiments demonstrated the relationship between ATF4 expression levels and apoptosis in asthma airway epithelial cells. **(A)** Protocol for Establishing an Asthma Mouse Model. **(B, C)** Western blot analysis of ATF4 protein expression in mouse lung tissue and statistical analysis (n=3). **(D)** Scanning electron microscopy examination of damage to mouse bronchial epithelium (n=4). **(E, F)** TUNEL assay for apoptosis in mouse bronchial epithelial cells and statistical analysis (n=4). **(G, H)** Western blot analysis of ATF4 protein expression in BEAS-2B cells and statistical analysis (n=3). **(I, M, N)** Annexin V-FITC/PI double staining to assess apoptosis in BEAS-2B cells and statistical analysis (n=4). **(G, K, L)** Cell scratch assay to assess the migratory capacity of BEAS-2B cells and statistical analysis (n=4).

To further investigate the relationship between ATF4 and apoptosis in asthma airway epithelial cells, we used lentiviral transfection to knock down ATF4 in BEAS-2B cells. On day 4 post-transfection, GFP expression was examined by confocal microscopy (**Figure 2A**). Flow cytometric analysis revealed that the proportion of GFP-positive BEAS-2B cells exceeded 85% (**Figure 2B**), confirming successful lentiviral transfection. Subsequent qPCR (*P* < 0.001) and Western blot (*P* < 0.01) analyses demonstrated that the PSC60302 lentivirus strain produced the most effective knockdown (**Figures 2C–E**); therefore, stable cell lines derived from this virus were used in all subsequent experiments. Apoptosis was assessed using Annexin V-Cy5 staining (**Figures 2F, G**). Compared with the LV-GFP control group, the red fluorescence intensity (indicative of apoptosis) was significantly higher in the LV-GFP+HDM group (*P* < 0.001), indicating that HDM treatment increased apoptosis in BEAS-2B cells. Compared with the LV-GFP+HDM group, the LV-shATF4+HDM group exhibited reduced red fluorescence intensity (*P* < 0.001), indicating that ATF4 knockdown attenuates HDM-induced apoptosis in BEAS-2B cells. Results from the cell scratch assay (**Figures 2H–J**) showed that the LV-shATF4+HDM group exhibited significantly enhanced scratch healing capacity compared with the LV-GFP+HDM group at both 24h and 48h (*P* < 0.05). These findings indicate that ATF4 expression is positively correlated with epithelial cell injury and apoptosis, whereas ATF4 knockdown reduces injury and apoptosis and promotes migration and repair in asthma airway epithelial cells.

**Figure 2:**
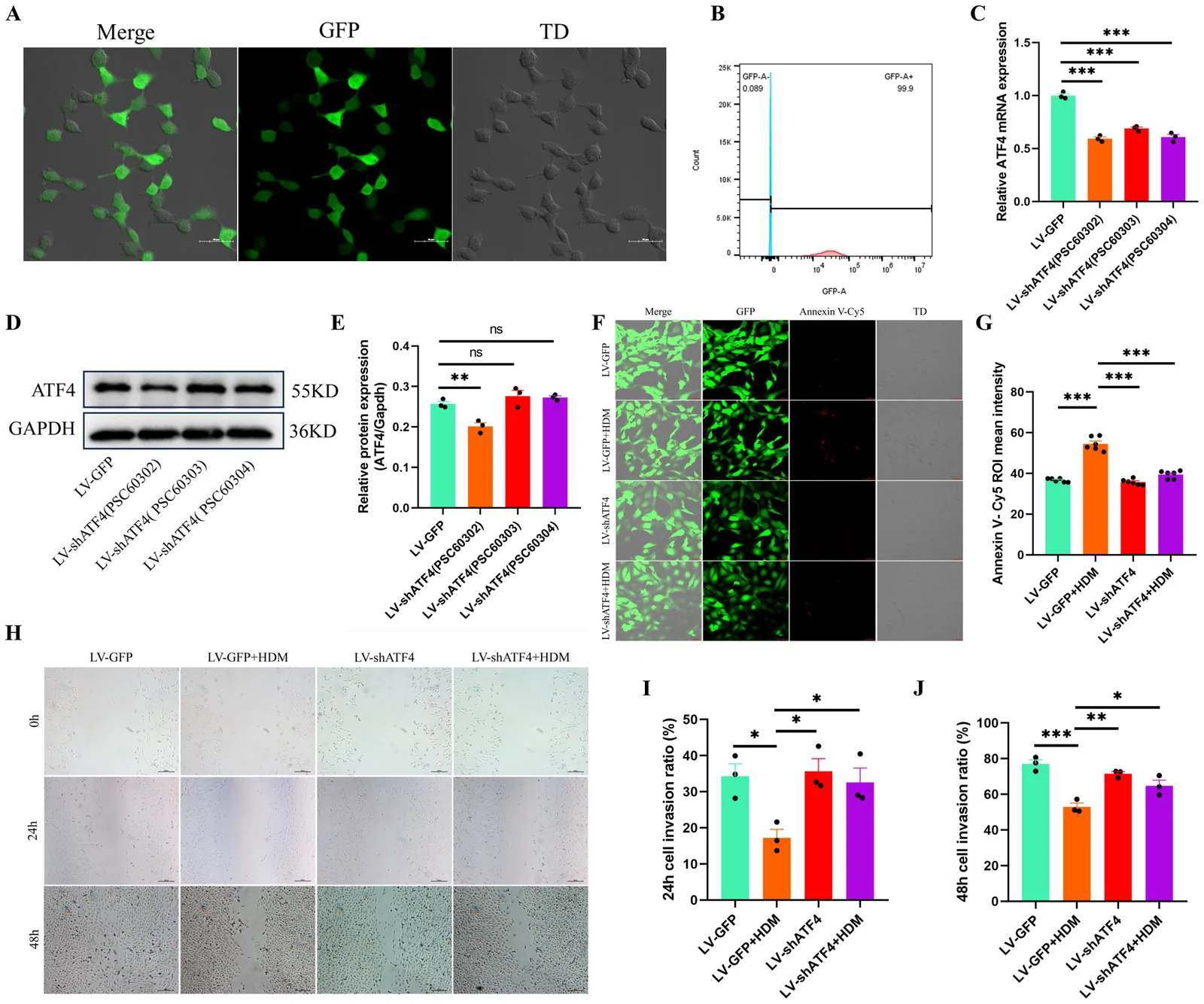
Investigating the effects of knocking down ATF4 in BEAS-2B cells on apoptosis and migratory capacity. **(A)** GFP expression following lentiviral transfection, as observed by laser confocal microscopy. **(B)** Proportion of GFP-positive cells following transfection, as determined by flow cytometry. **(C)** qPCR validation of ATF4 knockdown (n=3). **(D, E)** Western blot validation of ATF4 knockdown (n=3). **(F, G)** Annexin V-Cy5 detection of apoptosis in BEAS-2B cells following ATF4 knockdown and statistical analysis (n=6). **(H–J)** Cell scratch assay to assess the migratory capacity of BEAS-2B cells following ATF4 knockdown and statistical analysis (n=3).

### ATF4 may regulate mitochondrial stress damage via the ATF4-CHOP-Bcl-2 signaling pathway, thereby mediating epithelial cell apoptosis

To investigate the potential mechanisms by which ATF4 mediates damage and apoptosis in asthma airway epithelial cells, total RNA was extracted from BEAS-2B cells in the LV-GFP+HDM and LV-shATF4 groups and submitted for RNA sequencing. Venn diagram analysis showed that the LV-shATF4 group and the LV-GFP+HDM group shared 13,103 genes, with 533 genes specific to the LV-shATF4 group and 446 genes specific to the LV-GFP+HDM group (**Figure 3A**). A total of 214 differentially expressed genes were identified (**Figures 3B, C**), including 117 upregulated and 97 downregulated genes. GO enrichment analyses were then performed on the upregulated and downregulated gene sets (**Figures 3D, E**). The downregulated gene set was significantly enriched in biological processes related to apoptosis, indicating that ATF4 knockdown reduces the expression of apoptosis-related genes. To further investigate potential ATF4-interacting proteins, a protein–protein interaction network was constructed using the STRING database, which revealed that ATF4 potentially interacts with DDIT3 (also known as CHOP) and Bcl2 (**Figure 3F**). CHOP plays a crucial role in various cellular responses (such as mitochondrial stress and endoplasmic reticulum stress) and induces cell cycle arrest and apoptosis, primarily through ATF4-dependent transcriptional induction (15, 16). The Bcl-2 gene encodes a complete mitochondrial membrane protein whose primary function is to regulate the opening of the mitochondrial permeabilization transition pore (mPTP), thereby controlling the process of apoptosis (17–19). Based on the above analysis and supporting literature, we propose that ATF4 may mediate mitochondrial stress damage by regulating the ATF4–CHOP–Bcl2 signaling axis, thereby influencing apoptosis in airway epithelial cells.

**Figure 3:**
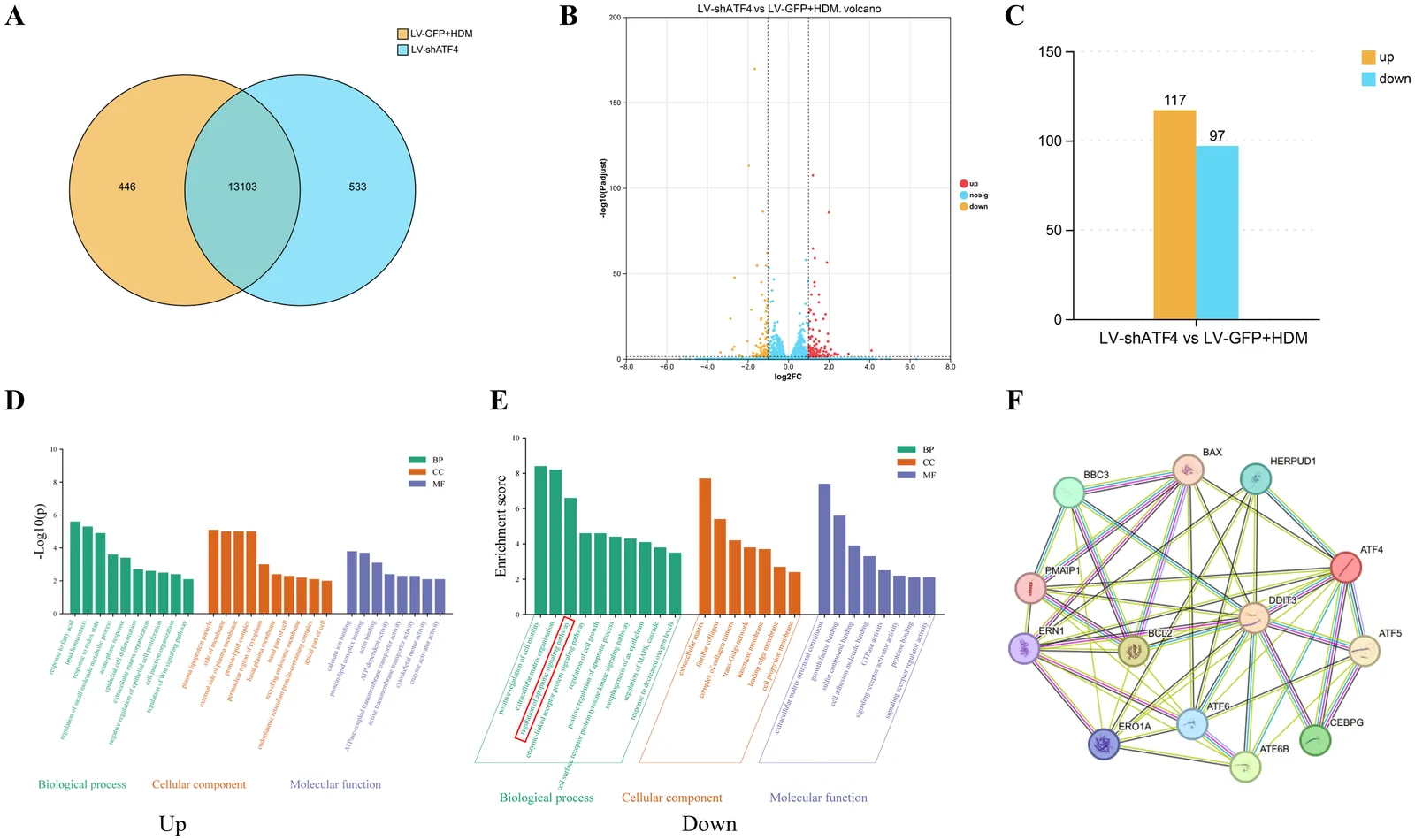
RNA sequencing and biological analysis (n=3). **(A)** Venn diagram **(B)** Volcano plot of differentially expressed genes. **(C)** Bar chart of differentially expressed genes **(D)** GO enrichment analysis of upregulated differentially expressed genes. **(E)** GO enrichment analysis of downregulated differentially expressed genes. **(F)** PPI analysis of ATF4.

### Knockdown of ATF4 may alleviate mitochondrial stress damage in epithelial cells by regulating the ATF4-CHOP-Bcl2 signaling pathway

To investigate the effects of ATF4 knockdown on mitochondria in BEAS-2B cells, MitoSox Red was used to label mitochondrial superoxide. As shown in **Figures 4A, C**, mitochondrial ROS fluorescence intensity was increased in the LV-GFP+HDM group compared with the LV-GFP control group (*P* < 0.001), indicating that HDM treatment enhanced mitochondrial oxidative stress. In contrast, mitochondrial ROS fluorescence intensity was significantly reduced in the LV-shATF4+HDM group compared with the LV-GFP+HDM group (*P* < 0.001), indicating that ATF4 knockdown reduces HDM-induced mitochondrial ROS elevation in BEAS-2B cells. Mitochondrial membrane potential was assessed using TMRE (**Figures 4B, D**). TMRE fluorescence intensity was significantly reduced in the LV-GFP+HDM group compared with the LV-GFP control group (*P* < 0.001), indicating that HDM treatment decreased mitochondrial membrane potential in BEAS-2B cells. In contrast, TMRE fluorescence intensity was markedly restored in the LV-shATF4+HDM group compared with the LV-GFP+HDM group (*P* < 0.001), indicating that ATF4 knockdown reverses HDM-induced mitochondrial membrane potential loss in BEAS-2B cells. Western blot analysis was performed to assess the expression levels of the mitochondrial fission proteins Drp1 and p-Drp1, the fusion protein Mfn2, and the mitochondrial functional marker protein ATP5F1A in BEAS-2B cells from each group (**Figures 4E, G–J**). Compared with the LV-GFP control group, the LV-GFP+HDM group showed significantly increased expression of p-Drp1 and total Drp1 (both *P* < 0.01), and significantly reduced expression of Mfn2 and ATP5F1A (both *P* < 0.01). The findings indicate that HDM treatment induces mitochondrial damage, characterized by enhanced fission, reduced fusion, and impaired mitochondrial function. Compared with the LV-GFP+HDM group, the LV-shATF4+HDM group exhibited significantly reduced expression of p-Drp1 and Drp1 (both *P* < 0.05) and significantly increased expression of Mfn2 and ATP5F1A (both *P* < 0.001). These results suggest that ATF4 knockdown inhibits HDM-induced excessive mitochondrial fission, promotes fusion, and alleviates mitochondrial dysfunction. Transmission electron microscopy was used to examine mitochondrial ultrastructure in BEAS-2B cells (**Figure 4F**). Mitochondria in the LV-GFP control group exhibited typical rod-like morphology with clearly visible cristae and intact structure. In contrast, mitochondria in the LV-GFP+HDM group showed obvious swelling and blurred or absent cristae, indicating HDM-induced structural damage. Mitochondrial morphology in the LV-shATF4+HDM group was significantly improved, with identifiable cristae and no obvious swelling or shrinkage, indicating that ATF4 knockdown alleviates HDM-induced mitochondrial structural abnormalities. The above results indicate that knocking down ATF4 in BEAS-2B cells can mitigate mitochondrial stress damage in BEAS-2B cells following HDM treatment.

**Figure 4:**
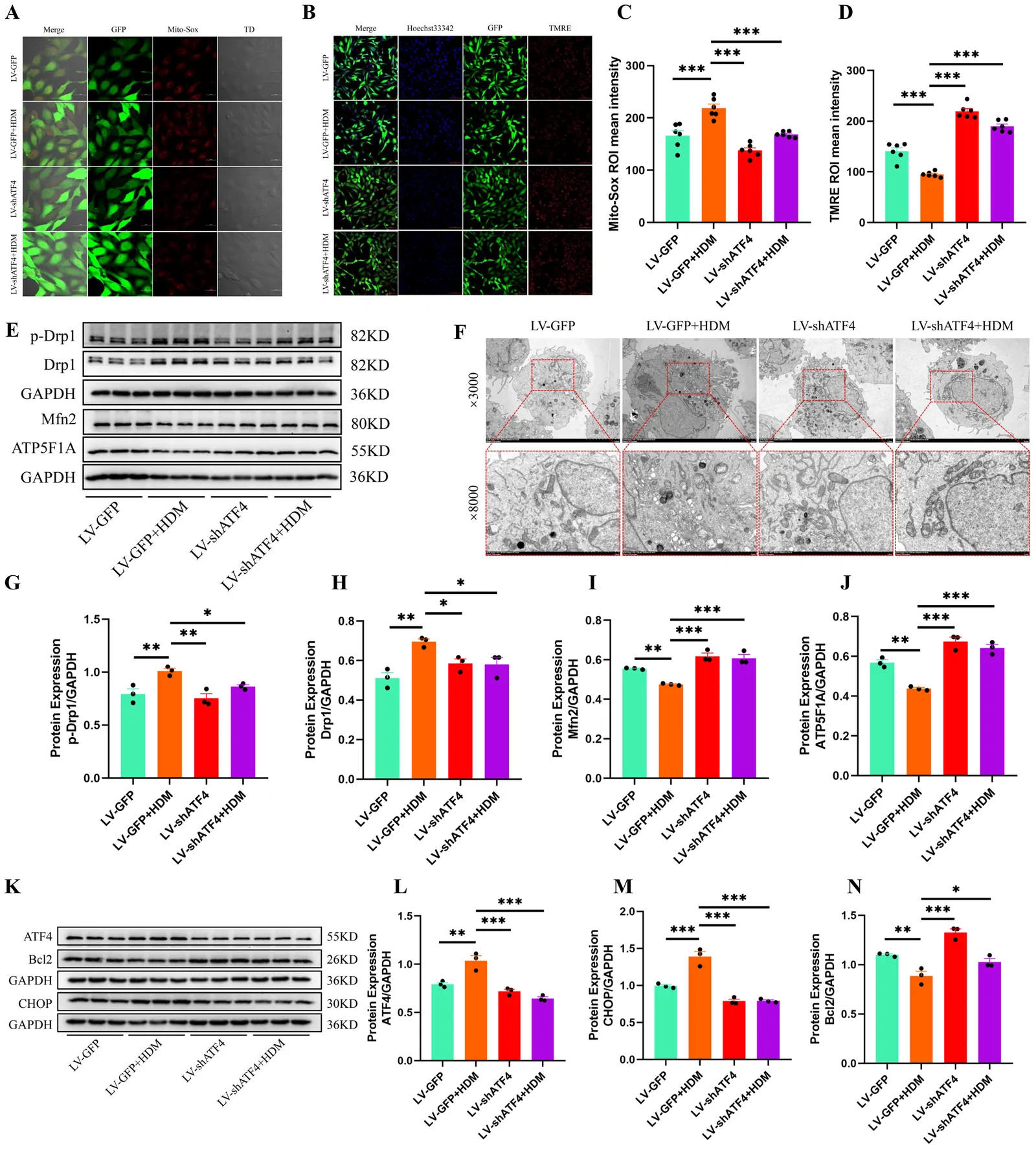
Effects of ATF4 knockdown on mitochondria and pathway proteins in BEAS-2B cells. **(A, C)** MitoSox Red assay for mitochondrial reactive oxygen species levels (n=6). **(B, D)** TMRE assay for mitochondrial membrane potential (n=6). **(E, G–J)** Western blot detection of mitochondrial damage-associated protein expression in BEAS-2B cells and statistical analysis (n=3). **(F)** Transmission electron microscopy analysis of mitochondrial ultrastructure (n=3). **(K–N)** Western blot results of the expression of proteins in the ATF4-CHOP-Bcl2 signaling pathway and statistical analysis (n=3).

The bioinformatics analysis described above suggests that ATF4 may mediate mitochondrial stress damage via the ATF4–CHOP–Bcl2 signaling axis, thereby influencing cellular damage and apoptosis. Subsequent experiments confirmed that ATF4 knockdown alleviates HDM-induced mitochondrial stress damage and apoptosis in airway epithelial cells. To further verify whether ATF4 regulates these processes via the ATF4–CHOP–Bcl2 signaling axis, Western blot analysis was performed to detect the levels of ATF4, CHOP, and Bcl2 in BEAS-2B cells from each group (**Figures 4K–N**). Compared with the LV-GFP control group, the LV-GFP+HDM group exhibited significantly increased expression of ATF4 (*P* < 0.01) and CHOP (*P* < 0.001), and significantly decreased expression of Bcl2 (*P* < 0.01). These findings indicate that HDM treatment activates the ATF4–CHOP–Bcl2 signaling axis. Compared with the LV-GFP+HDM group, the LV-shATF4+HDM group showed significantly reduced expression of ATF4 and CHOP (both *P* < 0.001), whereas Bcl2 expression was significantly restored (*P* < 0.05). These results indicate that ATF4 knockdown effectively suppresses HDM-induced CHOP upregulation and restores Bcl2 expression, supporting the conclusion that ATF4 mediates mitochondrial stress-induced damage and apoptosis in airway epithelial cells through the ATF4–CHOP–Bcl2 signaling axis.

### NG-R1 improves ventilatory function in asthmatic mice and reduces pulmonary inflammation

The drug treatment protocol for each group of mice is shown in **Figure 5A**. Noninvasive pulmonary function testing was used to measure changes in lung function in each group of mice following inhalation of increasing concentrations of methacholine. The Penh value was used as an indicator of airway responsiveness. As shown in **Figure 5B**, Penh values increased significantly in a dose-dependent manner in the asthma group compared with the control group. At acetylmethcholine nebulization concentrations of 6.25 mg/mL or higher, the Penh values in the NG-R1 group were significantly lower than those in the asthma group (*P* < 0.05), indicating that NG-R1 improves lung ventilation, reduces airway hyperresponsiveness, and alleviates airway resistance in asthmatic mice.

**Figure 5:**
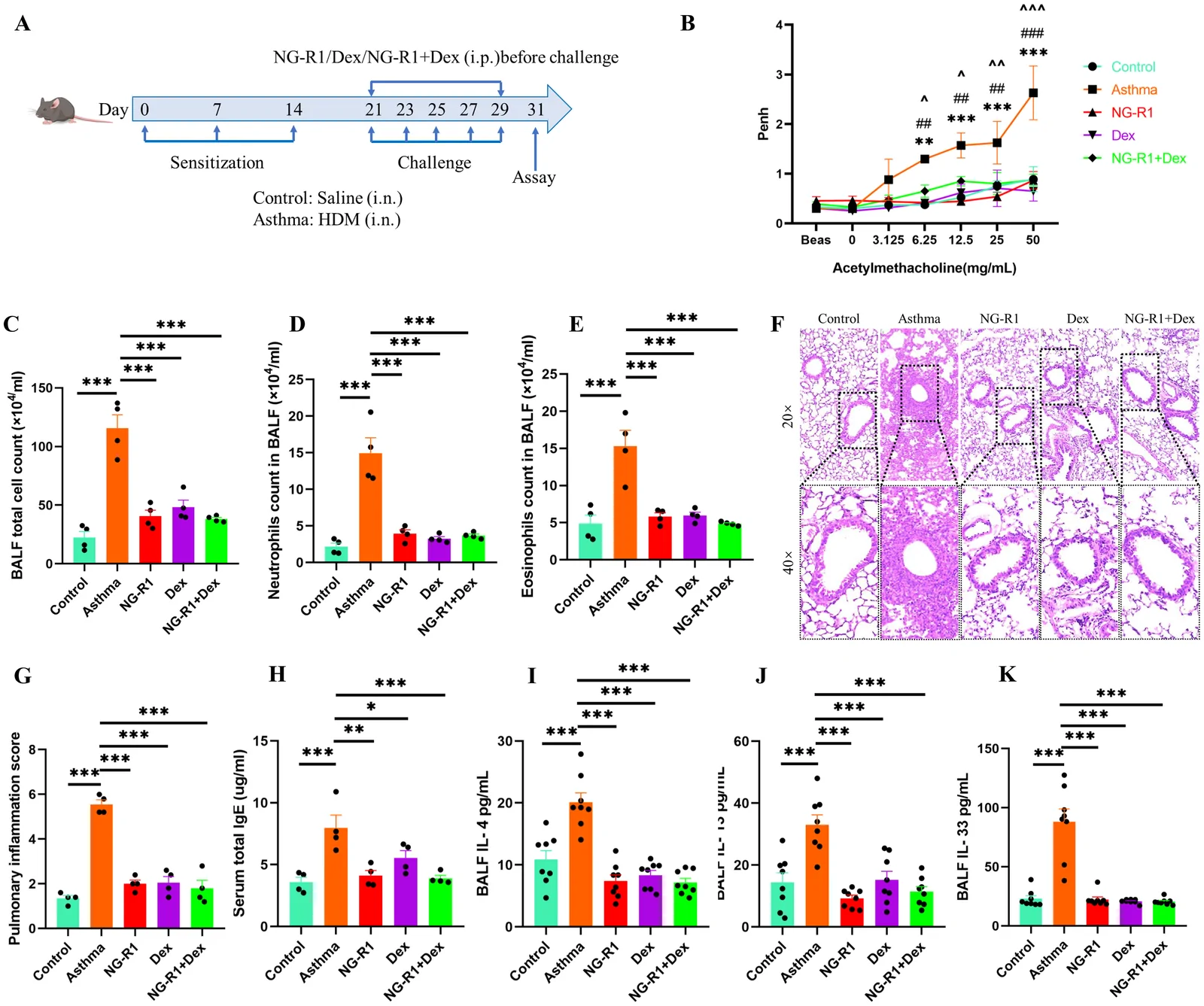
Therapeutic effects of NG-R1 on pulmonary inflammation in asthmatic mice. **(A)** Intervention protocol for asthmatic mice. **(B)** Lung function testing in mice (n=4). **(C–E)** Total and differential cell counts in mouse BALF (n=4). **(F, G)** H&E staining of mouse lung tissue and pathological scoring of pulmonary inflammation (n=4). **(H)** ELISA assay for serum IgE levels in mice (n=4). **(I–K)** ELISA assay for IL-4, IL-13, and IL-33 levels in mouse BALF (n=8).

Total and differential cell counts were performed on BALF to assess pulmonary inflammatory cell infiltration in each group. As shown in **Figures 5C–E**, total cell, neutrophil, and eosinophil count in BALF were significantly higher in the asthma group than in the control group (all *P* < 0.001). In contrast, these counts were significantly lower in the NG-R1 group than in the asthma group (all *P* < 0.001), the NG-R1+Dex group, the total number of cells in BALF and the number of eosinophils decreased even more significantly (all *P* < 0.001), indicating that NG-R1 effectively reduces the levels of inflammatory cells in the lungs of asthmatic mice, and when used in combination with Dex, it further reduces the levels of inflammatory cells in the BALF of asthmatic mice.

H&E staining was used to assess airway epithelial morphology and peribronchial inflammatory cell infiltration in lung tissue from each group. As shown in **Figures 5F, G**, the asthma group exhibited extensive peribronchial inflammatory cell infiltration, along with varying degrees of airway epithelial desquamation and disruption, compared with the control group. In contrast, following NG-R1 intervention, pulmonary inflammation was markedly reduced, and the bronchial epithelium remained relatively intact with no obvious desquamation. Pathological scoring of pulmonary inflammation confirmed that the inflammation score was significantly lower in the NG-R1 group than in the asthma group (*P* < 0.001), indicating that NG-R1 effectively reduces pulmonary inflammatory infiltration and preserves airway epithelial integrity in asthmatic mice.

Total serum IgE levels were measured by ELISA (**Figure 5H**). Compared with the control group, total serum IgE levels were significantly elevated in the asthma group (*P* < 0.001), indicating successful sensitization and a Th2-biased immune response *in vivo*. Compared with the asthma group, serum IgE levels were significantly reduced in the NG-R1 group (*P* < 0.01). BALF supernatant was analyzed by ELISA to determine the levels of the Th2 cytokines IL-4, IL-13, and IL-33 (**Figures 5I–K**). Compared with the control group, IL-4, IL-13, and IL-33 levels in BALF were significantly elevated in the asthma group (all *P* < 0.001). Following NG-R1 intervention, the levels of these cytokines were significantly lower than those in the asthma group (all *P* < 0.001), and the combined use of NG-R1 and Dex resulted in a more pronounced reduction in IL-4 and IL-33 levels in the BALF of asthmatic mice (both *P* < 0.001). In summary, NG-R1 effectively suppresses the expression of Th2 cytokines in the lungs of asthmatic mice and attenuates the Th2 immune response; when combined with Dex, it further reduces the inflammatory response in the lungs of asthmatic mice.

### NG-R1 reduces mitochondrial stress damage and epithelial cell apoptosis in asthmatic mice airway epithelium

Western blot analysis was performed to assess the expression levels of the mitochondrial fission proteins Drp1 and p-Drp1, the fusion protein Mfn2, and the mitochondrial functional marker protein ATP5F1A in mice lung tissues from each group (**Figures 6A–E**). Compared with the control group, the asthma group exhibited significantly increased p-Drp1 expression and p-Drp1/Drp1 ratio (both *P* < 0.05), and significantly decreased Mfn2 (*P* < 0.05) and ATP5F1A (*P* < 0.001) expression. Compared with the asthma group, the NG-R1 group showed significantly reduced p-Drp1 expression and p-Drp1/Drp1 ratio (both *P* < 0.01) and significantly restored Mfn2 and ATP5F1A expression (both *P* < 0.05). These findings indicate that NG-R1 effectively inhibits excessive mitochondrial fission in the lungs of asthmatic mice, promotes fusion, restores mitochondrial function, and alleviates mitochondrial damage. Transmission electron microscopy was used to examine mitochondrial ultrastructure in bronchial epithelial cells from mice in each group (**Figure 6F**). Compared with the control group, mitochondria in bronchial epithelial cells of the asthmatic mice showed significant damage, characterized by swelling and blurred or absent cristae. Following NG-R1 intervention, mitochondrial morphology improved significantly compared with the asthma group, returning to a normal rod-like shape, which indicating that NG-R1 effectively ameliorates mitochondrial ultrastructural abnormalities in asthma airway epithelial cells. Apoptosis in airway epithelial cells was assessed using TUNEL staining (**Figures 6G, I**). Compared with the control group, the asthmatic mice exhibited significant TUNEL-positive red fluorescence signals in bronchial epithelial cells (*P* < 0.001). Compared with the asthma group, TUNEL fluorescence intensity was significantly reduced in the NG-R1 group (*P* < 0.001), indicating that NG-R1 effectively inhibits airway epithelial cell damage and apoptosis in asthmatic mice. Scanning electron microscopy was used to examine the surface structure and ciliary morphology of bronchial epithelial cells in mice (**Figure 6H**). Compared with the control group, bronchial epithelial cilia in the asthma group showed significant thinning, shortening, shedding, and defects. The airway surface was also covered with abundant mucus-like secretions, leading to ciliary collapse. Following NG-R1 intervention, ciliary structure was significantly improved compared with the asthma group, and no obvious flattening or shedding was observed, and airway surface mucus secretion was minimal. *In vivo* experimental results indicate that NG-R1 effectively reduces mitochondrial damage in bronchial epithelial cells of asthmatic mice and alleviates bronchial epithelial cell apoptosis.

**Figure 6:**
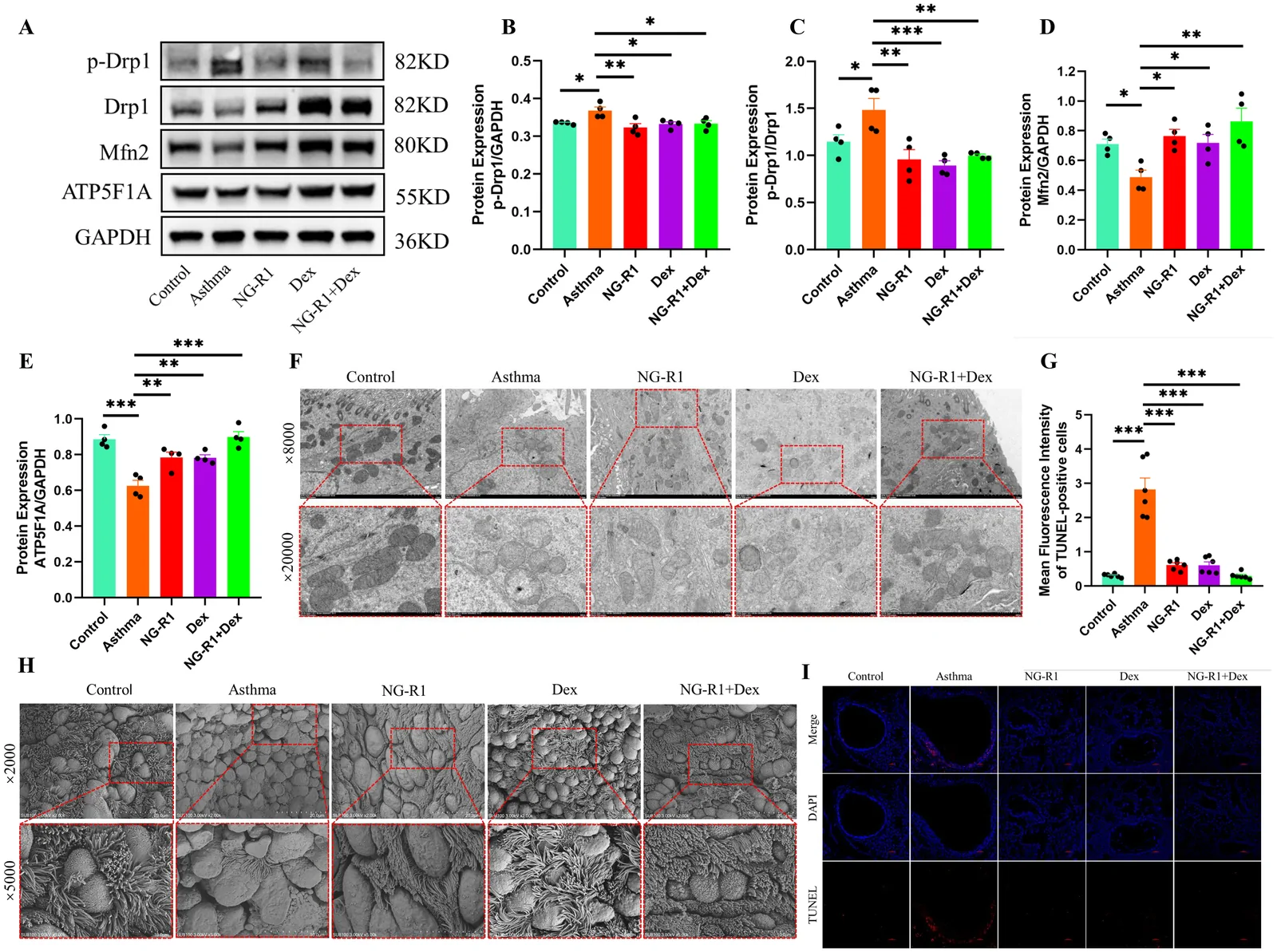
NG-R1 alleviates mitochondrial stress damage to the asthmatic mice airway epithelium, as well as epithelial cell damage and apoptosis. **(A–E)** Western blot results of the expression of proteins associated with mitochondrial damage in asthmatic mice lung tissue and statistical analysis (n=4). **(F)** Examination of the morphology and ultrastructure of mitochondria in bronchial epithelial cells of asthmatic mice using transmission electron microscopy (n=3). **(H)** Scanning electron microscopy analysis of bronchial epithelial integrity in asthmatic mice (n=3). **(G, I)** TUNEL assay for apoptosis in bronchial epithelial cells of asthmatic mice (n=6).

BEAS-2B cells were cultured *in vitro* and divided into control, HDM, NG-R1, Dex, and combined NG-R1+Dex groups. Mitochondrial ROS were labeled using MitoSox Red fluorescent probes (**Figures 7A, B**). Compared with the control group, mitochondrial ROS fluorescence intensity was significantly increased in the HDM group (*P* < 0.001). Compared with the HDM group, the NG-R1 group showed a reduction in mitochondrial ROS fluorescence intensity (*P* < 0.001), the red fluorescence intensity in the NG-R1+Dex group decreased further (*P* < 0.001), indicating that NG-R1 effectively reduces HDM-induced mitochondrial ROS levels, combining it with Dex can further alleviate mitochondrial oxidative stress in BEAS-2B cells. JC-1 staining was used to assess mitochondrial membrane potential (**Figures 7C, D**). Compared with the control group, the HDM group showed reduced red fluorescence and enhanced green fluorescence, resulting in a significantly lower red-to-green fluorescence ratio (*P* < 0.001), indicating that HDM stimulation decreases mitochondrial membrane potential in BEAS-2B cells. Compared with the HDM group, the NG-R1 group showed enhanced red fluorescence and weakened green fluorescence, with a recovery in the red-to-green fluorescence ratio (*P* < 0.001), and the red-to-green fluorescence ratio was significantly higher in the NG-R1+Dex group (*P* < 0.001), indicating that NG-R1 restores HDM-induced mitochondrial membrane potential decrease in BEAS-2B cells, and when used in combination with Dex, it can further restore mitochondrial membrane potential levels. Western blot analysis was performed to assess the expression levels of the mitochondrial fission proteins Drp1 and p-Drp1, the fusion protein Mfn2, and the mitochondrial functional marker protein ATP5F1A in BEAS-2B cells from each group (**Figures 7E–I**). Compared with the control group, the HDM group showed significantly increased p-Drp1 (*P* < 0.05) and total Drp1 (*P* < 0.001) expression, and significantly decreased Mfn2 (*P* < 0.001) and ATP5F1A (*P* < 0.05) expression. Compared with the HDM group, the NG-R1 group showed reduced p-Drp1 (*P* < 0.001) and Drp1 (*P* < 0.01) expression, and increased Mfn2 (*P* < 0.01) and ATP5F1A (*P* < 0.05) expression. These findings indicate that NG-R1 effectively inhibits HDM-induced excessive mitochondrial fission, promotes fusion, improves mitochondrial function, and alleviates mitochondrial stress damage. Transmission electron microscopy (**Figure 7J**) revealed that, compared with the control group, mitochondria in the HDM group exhibited significant morphological abnormalities, including swelling and blurred or absent cristae. In contrast, mitochondria in the NG-R1 group largely returned to a normal rod-like morphology with clearly visible cristae. Apoptosis was assessed using Annexin V-FITC/PI double staining (**Figure 7K, L, P**). Compared with the control group, the mean fluorescence intensity of both Annexin V-FITC-positive and PI-positive cells was significantly higher in the HDM group (both *P* < 0.001). Compared with the HDM group, the fluorescence intensities of both Annexin V-FITC and PI were significantly lower in the NG-R1 group (both *P* < 0.001), indicating that NG-R1 effectively reduces HDM-induced apoptosis in BEAS-2B cells. The cell scratch assay (**Figures 7M–O**) showed that, compared with the control group, the HDM group exhibited significantly poorer wound healing and reduced cell migration rates at both 24h and 48h post-scratch (both *P* < 0.001). Compared with the HDM group, NG-R1-treated cells showed significantly enhanced scratch healing at both time points; by 48h, wounds had nearly completely closed. Migration rates at 24h and 48h were significantly higher than those in the HDM group (both *P* < 0.001). The *in vitro* results demonstrate that NG-R1 effectively alleviates HDM-induced mitochondrial stress damage, reduces apoptosis, and promotes the migration and repair of damaged epithelial cells.

**Figure 7:**
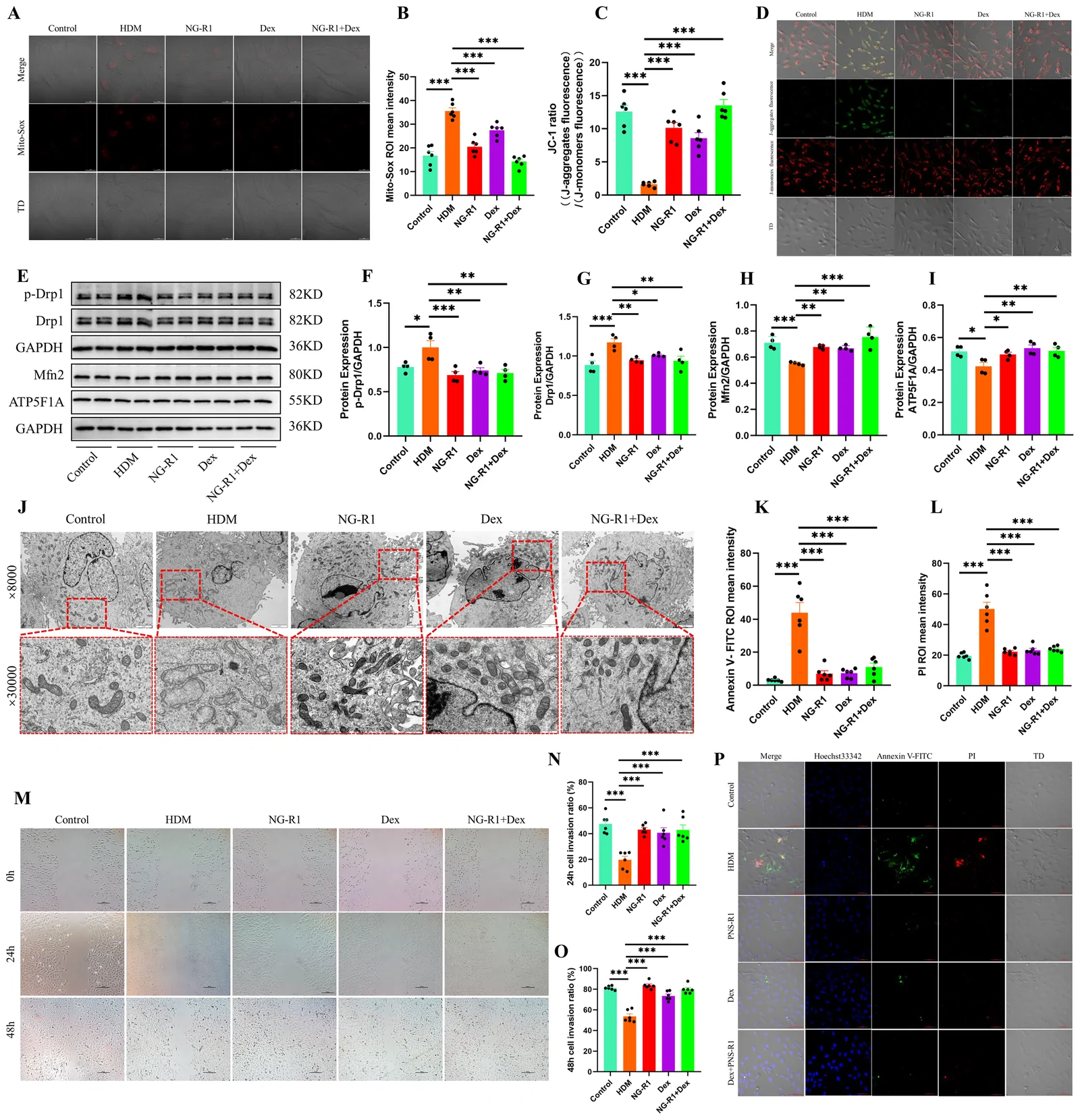
NG-R1 alleviates mitochondrial stress damage and apoptosis in BEAS-2B cells. **(A, B)** MitoSox Red assay for mitochondrial reactive oxygen species levels in BEAS-2B cells (n=6). **(C, D)** JC-1 assay for mitochondrial membrane potential in BEAS-2B cells (n=6). **(E–I)** Western blot results of the expression of mitochondrial damage-related proteins in BEAS-2B cells and statistical analysis (n=4). **(J)** Examination of the morphology and ultrastructure of mitochondria in BEAS-2B cells by transmission electron microscopy (n=3). **(K, L, P)** Annexin V-FITC/PI staining to assess apoptosis in BEAS-2B cells (n=6). **(M–O)** Cell scratch assay to assess the migratory capacity of BEAS-2B cells (n=6).

### NG-R1 may target ATF4 for downregulation, modulate the ATF4-CHOP-Bcl2 signaling pathway to alleviate mitochondrial stress damage, and reduces apoptosis in epithelial cells

To investigate whether NG-R1 alleviates mitochondrial damage and epithelial cell apoptosis by targeting ATF4 and thereby regulating the ATF4–CHOP–Bcl2 signaling pathway, molecular docking analyses were conducted to evaluate the binding patterns of NG-R1 with mouse and human ATF4 proteins (**Figures 8A, B**). In the docking of NG-R1 with mouse ATF4, NG-R1 formed strong hydrophobic interactions with residues ALA-299, PHE-177, GLU-300, ALA-303, and LEU-304, which helped stabilize the small molecule within the receptor’s hydrophobic pocket. Simultaneously, NG-R1 formed multiple hydrogen bonds with residues GLU-307, LYS-296, GLU-174, GLN-295, and ARG-292, further stabilizing the complex structure. In the docking of NG-R1 with human ATF4, NG-R1 formed significant hydrophobic interactions with residues TYR-295, ARG-300, GLN-303, and LYS-299, enhancing intermolecular affinity. Additionally, multiple hydrogen bonds formed with residues TRP-84, ARG-300, GLN-303, and ARG-296, further stabilizing the complex conformation. Analysis of the polar interactions at the binding site between NG-R1 and ATF4 indicates that the stability of this binding site depends primarily on the synergistic action of hydrophobic interactions and hydrogen bonds. Furthermore, the binding energies of NG-R1 with mouse and human ATF4 were −5.761kcal/mol and −6.553kcal/mol, respectively, both below −5kcal/mol, indicating stable binding between NG-R1 and ATF4 (**Table 3**).

**Figure 8:**
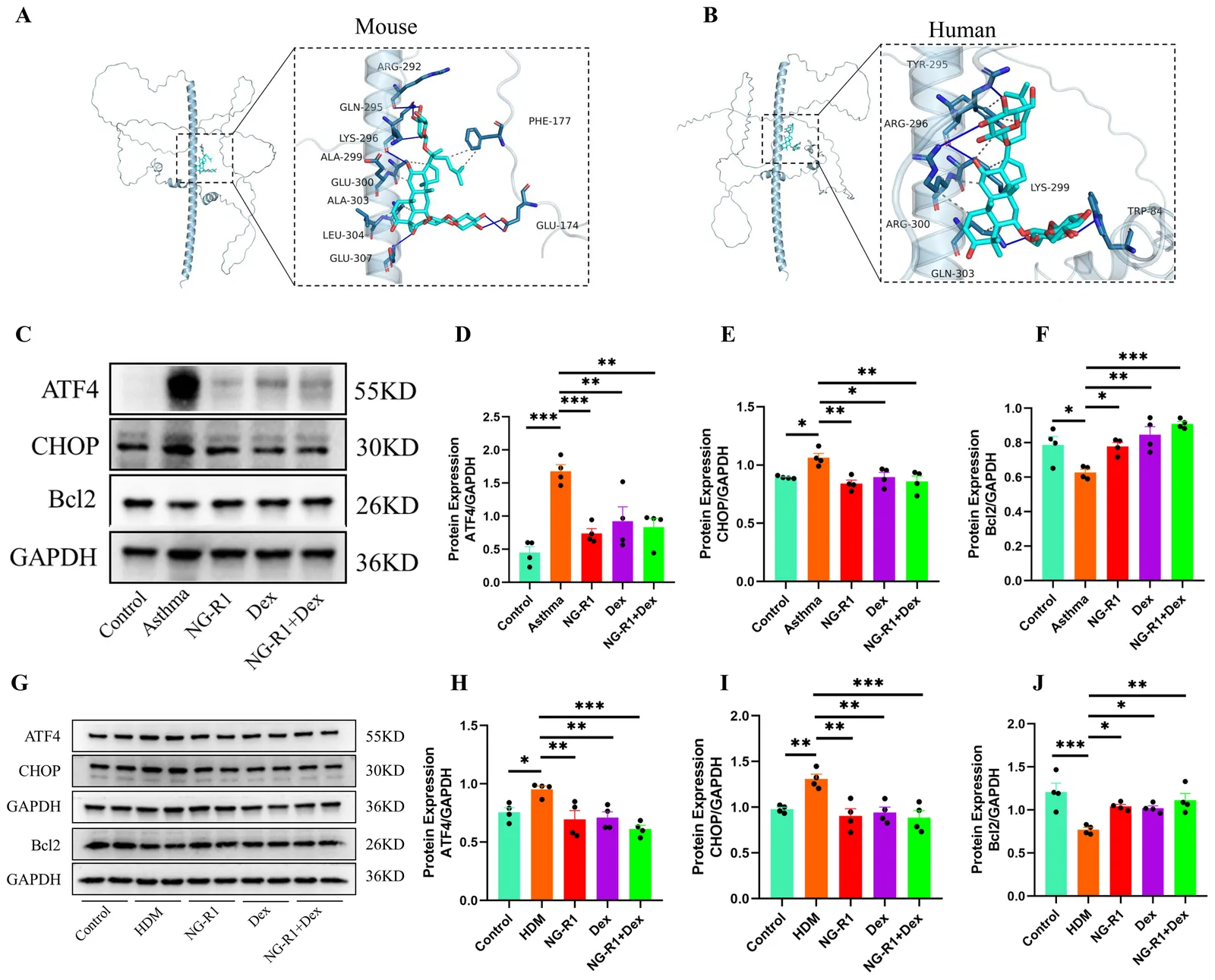
Molecular docking of NG-R1 with ATF4 and Western blot analysis to validate the expression of pathway proteins. **(A, B)** Molecular docking of NG-R1 with mouse and human ATF4 proteins. **(C–F)** Western blot results of ATF4-CHOP-Bcl2 pathway protein expression in lung tissue from asthmatic mice and statistical analysis (n=4). **(G–J)** Western blot results of ATF4-CHOP-Bcl2 pathway protein expression in BEAS-2B cells and statistical analysis (n=4).

**Table 3:** Binding energy.

Species	Target name	Ligand name	Docking score (kcal/mol)
Mouse	ATF4	NG-R1	-5.761
Human	ATF4	NG-R1	-6.553

Western blot analysis was performed to detect the expression levels of ATF4, CHOP, and Bcl2 in mice lung tissues from each group (**Figures 8C–F**). Compared with the control group, the asthmatic mice showed significantly elevated expression of ATF4 (*P* < 0.001) and CHOP (*P* < 0.05), and reduced expression of the anti-apoptotic protein Bcl2 (*P* < 0.05). Compared with the asthma group, the NG-R1 group exhibited significantly downregulated expression of ATF4 (*P* < 0.001) and CHOP (*P* < 0.01), and significantly restored Bcl2 expression (*P* < 0.05). Similarly, total proteins were extracted from BEAS-2B cells in each group and analyzed by Western blot. As shown in **Figures 8G–J**, compared with the control group, the HDM-treated group showed significantly upregulated expression of ATF4 and CHOP (both *P* < 0.05), and markedly downregulated expression of Bcl2 (*P* < 0.001). Following NG-R1 treatment, the HDM-induced upregulation of ATF4 (*P* < 0.01) and CHOP (*P* < 0.01) was significantly reversed, and Bcl2 expression was restored (*P* < 0.05). The results of the *in vivo* and *in vitro* experiments described above indicate that NG-R1 may reduce apoptosis in asthmatic airway epithelial cells and promotes epithelial cell migration and repair by downregulating ATF4 expression and modulating the ATF4-CHOP-Bcl2 signaling axis to alleviate mitochondrial stress-induced damage.

## Discussion

As the body’s first line of defense against the external environment, the airway epithelium performs multiple functions essential for maintaining respiratory homeostasis, including gas and fluid transport, oxidative defense, ciliary clearance, and antimicrobial protection (20). When exposed to external stimuli such as allergens, pollutants, or viruses, the tight junctions between airway epithelial cells are disrupted, leading to increased barrier permeability. This facilitates allergen penetration across the epithelial barrier, more importantly, damaged epithelial cells themselves actively release alarmins, including thymic stromal lymphopoietin (TSLP), IL-25, and IL-33. These cytokines serve as the initial signals that initiate and polarize type 2 immune responses, playing a critical role in the triggering and amplification of airway inflammation in asthma (21). Chronic epithelial damage and abnormal repair constitute the pathological basis for airway remodeling (22). In the context of recurrent epithelial injury, epithelial cells may undergo epithelial-mesenchymal transition (EMT), transforming into mesenchymal-like cells with secretory and proliferative capabilities that directly contribute to airway wall thickening, fibroblast activation, and extracellular matrix deposition. This process not only constitutes the structural basis for the irreversible airflow limitation in asthma but also creates a persistent pro-inflammatory and reparative microenvironment that drives disease progression (23, 24). In summary, airway epithelial damage occurs throughout the course of asthma, from the initial disruption of barrier function and release of immune activation signals, through the sustained maintenance of chronic inflammation, to the eventual development of airway remodeling. During this process, the airway epithelium transforms from a passive victim of structural barrier damage into an active participant that drives immune regulation and disease progression. This deepened understanding provides a crucial theoretical basis for asthma treatment strategies that target epithelial cell damage.

Numerous studies have shown that the airway epithelium in asthma patients is under chronic stress due to prolonged exposure to external irritants, including allergens, gaseous pollutants, chemicals, medications, bacteria, and viruses. Oxidative stress can lead to tissue damage and dysregulation of cellular signaling, thereby promoting the onset and progression of various respiratory diseases (11, 25). Sustained oxidative stress leads to redox imbalance and direct cellular damage. Concurrently, chronic airway inflammation induces phenotypic changes in epithelial cells, promoting the secretion of cytokines and inflammatory mediators. This triggers a cascade of inflammatory responses, further exacerbating airway epithelial inflammation and tissue damage, and severely impairing airway epithelial barrier function (26). When airway epithelial cells are exposed to external stimuli, the lung tissue initiates adaptive regulatory mechanisms. Specifically, it modulates ATF4 expression through the integration of stress responses, thereby influencing the expression of downstream stress molecules to mediate adaptive responses (6, 27). When external stressors persist and exceed the cells’ compensatory capacity, they can also induce the expression of pro-apoptotic target genes, triggering cell death if the stress cannot be resolved (28). In this study, we demonstrated through *in vivo* and *in vitro* experiments that prolonged allergen exposure leads to high ATF4 protein expression and increased apoptosis in bronchial epithelial cells. Besides, according to a recent study (29), Mendelian randomization and machine learning analyses using the pediatric asthma genome confirmed that ATF4 is one of the genes that significantly influences childhood asthma. Another recent study has shown that IL-13 significantly upregulates ATF4 in airway epithelial cells, and that the IL-13/ATF4/S100P axis plays a key role in epithelial barrier dysfunction in eosinophilic asthma (30), thereby validating the clinical relevance of ATF4 in the asthma population’s genome. These findings all support the clinical relevance of ATF4 in the pathogenesis of asthma. Furthermore, ATF4 knockdown in bronchial epithelial cells reduced apoptosis, confirming that ATF4 plays a role in regulating apoptosis in asthmatic airway epithelial cells. In the pathophysiology of asthma, prolonged and repeated exposure to allergens and other environmental triggers leads to persistently elevated oxidative stress in airway epithelial cells, thereby activating the ISR pathway and upregulating ATF4 expression. Sustained ATF4 activation and aberrant regulation of its downstream signaling pathways ultimately exacerbate airway epithelial damage and apoptosis, driving the progression of airway inflammation and remodeling in asthma.

Current research suggests that impaired epithelial barrier function in airway epithelial cells due to oxidative stress is one of the key factors in the development of asthma. As mitochondria are among the most critical organelles, the disruption of mitochondrial homeostasis by oxidative stress is receiving increasing attention in the pathophysiology of asthma (7). As the central hubs of cellular energy metabolism, mitochondria not only supply ATP through oxidative phosphorylation but also maintain the stability and functional integrity of the mitochondrial network through finely tuned regulatory mechanisms, including the dynamic balance of fusion and fission, biogenesis, and mitophagy. They are therefore essential for cell survival and the maintenance of cellular homeostasis (31). In the pathological state of asthma, prolonged exposure of airway epithelial cells to environmental irritants leads to sustained mitochondrial oxidative stress. This, in turn, induces mitochondrial dysfunction, including a decrease in mitochondrial membrane potential, excessive production of reactive oxygen species, metabolic imbalance, and abnormal mitochondrial autophagy. These changes ultimately lead to airway epithelial cell damage and apoptosis, exacerbating airway inflammation and remodeling, and driving asthma progression (32). To investigate the potential mechanisms by which ATF4 regulates apoptosis in asthma airway epithelial cells and to determine whether it mediates apoptosis via mitochondrial stress, we performed RNA-seq and GO analysis. These analyses revealed that biological processes related to apoptosis were significantly enriched following ATF4 knockdown, clearly indicating that ATF4 participates in regulating apoptosis in these cells. Furthermore, PPI analysis revealed potential protein-protein interactions between ATF4, CHOP, and Bcl2. According to the literature, CHOP and Bcl2 are involved in regulating the mitochondrial stress response and mediating mitochondrial stress-induced damage and apoptosis (16, 17). Based on the above analysis and literature evidence, we hypothesized that ATF4 mediates mitochondrial stress damage by regulating the ATF4–CHOP–Bcl2 signaling axis, thereby influencing apoptosis in airway epithelial cells. Subsequently, we demonstrated that ATF4 knockdown in BEAS-2B cells reduced HDM-induced mitochondrial ROS levels and restored mitochondrial membrane potential. Using transmission electron microscopy and Western blot analysis, we confirmed that ATF4 knockdown alleviated HDM-induced mitochondrial morphological abnormalities and reduced the expression of mitochondrial damage markers (p-Drp1, Drp1, Mfn2, and ATP5F1A). Finally, Western blot analysis confirmed that ATF4 knockdown regulates mitochondrial stress-induced damage through the ATF4–CHOP–Bcl2 signaling axis, thereby influencing epithelial cell apoptosis. In summary, we have demonstrated that airway epithelial cells exhibit a stress response following prolonged exposure to external irritants, leading to elevated ATF4 expression. Persistent, unresolved high ATF4 expression results in mitochondrial stress, ultimately causing injury and apoptosis in airway epithelial cells. These findings provide new experimental evidence for a deeper understanding of the molecular mechanisms underlying airway epithelial damage in asthma and offer insights into asthma treatment strategies targeting the ATF4–CHOP–Bcl2 signaling axis.

Panax notoginseng (Burk.) F.H. Chen, a traditional Chinese medicinal herb also known as Sanqi or Sanchi in China, has been used for over 600 years. Modern research has revealed that the core pharmacologically active components of Sanqi, possess a variety of biological activities. Among these, compounds such as Notoginseng triterpenes and NG-R1 have been the most extensively studied. NG-R1 exhibits a variety of pharmacological effects, including the inhibition of oxidative damage, fibrosis, inflammatory responses, apoptosis, and endoplasmic reticulum stress, and shows broad application potential in cardiovascular protection, neuroprotection, and immunomodulation (13). Recent studies have demonstrated that NG-R1 pretreatment inhibits lipopolysaccharide (LPS)-induced degradation of inhibitor of nuclear factor κB alpha (IκBα) and activation of nuclear factor κB (NF-κB) and caspase-3, thereby reducing inflammatory responses and apoptotic damage in cardiomyocytes (33). In the cerebral ischemia model, NG-R1 alleviates astrocyte and microglial activation by inhibiting the TLR4/MyD88/NF-κB signaling pathway, thereby restoring blood-brain barrier integrity and reducing neuroinflammation (34). In addition, NG-R1 promotes angiogenesis following cerebral ischemia through the NAMPT-NAD-SIRT1 cascade and the Notch/VEGFR-2 signaling pathway (35). Owing to its multi-target, multi-pathway pharmacological properties, NG-R1 is gradually transcending the boundaries of traditional applications and demonstrating remarkable therapeutic potential in addressing major modern health challenges such as cardiovascular and cerebrovascular diseases, nerve damage repair, skeletal metabolism regulation, and anti-aging. Given the multifaceted pharmacological effects of NG-R1 and its ability to modulate oxidative stress and apoptosis, we investigated its therapeutic role in asthma through *in vivo* and *in vitro* experiments. In asthmatic mice, NG-R1 effectively reduced pulmonary inflammatory cell infiltration and Th2 cytokine levels and improved ventilatory function, indicating potent anti-inflammatory and immunomodulatory effects. Furthermore, through *in vivo* and *in vitro* experiments, we have demonstrated that NG-R1 can mitigate apoptosis caused by mitochondrial stress damage in asthmatic airway epithelial cells, thereby promoting epithelial migration and repair. Our studies have shown that knocking down ATF4 in bronchial epithelial cells alleviates mitochondrial stress damage and epithelial cell apoptosis via the ATF4-CHOP-Bcl2 pathway. Therefore, to determine whether NG-R1 can specifically downregulate ATF4, we performed molecular docking analysis and Western blot assays. The results showed that NG-R1 forms a stable complex with mouse and human ATF4 proteins through hydrophobic interactions and hydrogen bonding. Finally, Western blot analysis further confirmed that NG-R1 may regulate the ATF4-CHOP-Bcl2 signaling pathway by targeting and downregulating ATF4 expression. In summary, NG-R1 may target ATF4 and downregulate its expression, thereby regulating mitochondrial stress damage through the ATF4-CHOP-Bcl2 signaling pathway, ultimately alleviating damage and apoptosis in asthmatic airway epithelial cells.

This study inevitably has some limitations. Although our study confirms that NG-R1 may regulate mitochondrial stress and mediates epithelial cell apoptosis by targeting ATF4 and acting through the ATF4–CHOP–Bcl2 signaling axis, evidence of binding between NG-R1 and ATF4 based solely on molecular docking is insufficient. Further validation of their direct interaction is required using experimental methods such as molecular dynamics simulations, surface plasmon resonance (SPR), and cryo-electron microscopy (cryo-EM) structural analysis. In addition, we should also directly verify the function of each downstream component (such as CHOP and Bcl2 overexpression/knockdown rescue experiments). However, the exact molecular basis for ATF4 downregulation by NG-R1 has not yet been established. Subsequent studies using chromatin immunoprecipitation (ChIP) and protein stability analyses could help clarify this mechanism in greater detail. Additionally, in this study, we only knocked down ATF4 in BEAS-2B cells; future work should further explore ATF4 overexpression as well as knockdown and overexpression in mouse models to strengthen the logical rigor of our findings. It is also important to note whether NG-R1 can indirectly regulate ATF4 through upstream antioxidant or anti-inflammatory mechanisms. NG-R1 may reduce ATF4 expression through a dual mechanism: indirectly alleviating mitochondrial stress; potentially interacting directly with ATF4. Going forward, we should use MitoTEMPO to test indirect pathways and ATF4 rescue mutants to determine whether NG-R1 indirectly affects ATF4 by altering the upstream environment (antioxidant) or acts directly on ATF4 itself. There are still some experimental details that need improvement, such as replacing Penh measurement with invasive airway resistance measurement to achieve more precise lung function assessment; improving other epithelial barrier integrity molecular markers and experiments, such as tight junction-related proteins (ZO-1, occludin, claudin) and transepithelial electrical resistance (TEER); and increasing the number of replicate samples in animal experiments to make the experimental data more supportive and persuasive in drawing conclusions.

## Conclusion

This study reveals the link between ATF4 and apoptosis in asthma airway epithelial cells, confirming that airway epithelial cells exhibit high ATF4 expression following exposure to external stimuli. This, in turn, triggers apoptosis through the mediation of mitochondrial stress damage. Meanwhile, NG-R1, an active component of the traditional Chinese medicine Sanqi, may alleviate mitochondrial stress damage in asthma airway epithelial cells by targeting and downregulating ATF4, regulate the ATF4-CHOP-Bcl2 signaling pathway to alleviate mitochondrial stress damage in asthma airway epithelial cells, thereby reducing epithelial cell damage and apoptosis and restoring airway epithelial integrity. In summary, these findings provide a preliminary scientific basis for the use of NG-R1 in asthma treatment and highlight NG-R1 as a promising candidate for targeted asthma therapy, demonstrating significant potential for the clinical translation of NG-R1.

## Data Availability

The datasets presented in this study can be found in online repositories. The names of the repository/repositories and accession number(s) can be found below: PRJNA1521752 (Bioproject, NCBI).
