# Supplementary material for: Notoginsenoside R1 modulates the ATF4-CHOP-Bcl2 signaling pathway to alleviate mitochondrial stress damage and apoptosis in asthmatic airway epithelial cells
Source: Front Immunol. 2026 Sep 28;17:1861285. doi: 10.3389/fimmu.2026.1861285 (PMC13656254; doi:10.3389/fimmu.2026.1861285)
Supplement: Supplementary file 1 [file DataSheet1.pdf]

frontiers

The International Society for the Advancement of Cytometry (ISAC) has highlighted the importance of including comprehensive methodological information to ensure data reproducibility and reliability. In line with this, Frontiers in Immunology now requires authors to submit a checklist for manuscripts that involve flow or mass cytometry. This checklist helps standardize the reporting process, improving the quality and transparency of published data. By doing so, we support scientific progress, making it easier for other researchers to replicate and validate experiments.

This form should be submitted with any manuscripts using flow or mass cytometry.

Sample/specimen/material description

☐ Total blood

☐ PBMCs

☐ Organ digests

Other Beas-2B Cell Suspension

Did the samples suffer any treatment before or after incubation with the antibodies?

☐ Drug ___

☐ Cell permeabilization ___

☐ Dye ___

☐ Propidium iodine

☑ Not applicable

Other ___

Instrument and antibodies

Name of the Cytometer BD FACSCanto Flow Cytometer

**Antibodies and targets**<br>Green Fluorescent Protein	**Fluorochrome/ Metal**<br>GFP	**Catalog number/Company**<br>-
e.g. anti-CD4	FITC	Cat. XXX/ XXX ltd.

Data analyses

1. Name of the software FlowJo________________________________
2. Reference gating strategy in the manuscript or supplementary material

Gating strategy in (eg Figure X) Figure 2B______________________________
